# Development and Progression of Shoulder Lesions and Their Influence on Sow Behavior

**DOI:** 10.3390/ani12030224

**Published:** 2022-01-18

**Authors:** Tara Gaab, Emily Nogay, Meghann Pierdon

**Affiliations:** 1 New Bolton Center, Department of Clinical Studies, School of Veterinary Medicine, University of Pennsylvania, 382 West Street Road, Kennett Square, PA 19348, USA; tgaab@vet.upenn.edu; 2 Large Animal Services at Marysville, Department of Veterinary Preventative Medicine, College of Veterinary Medicine, The Ohio State University, 16410 County Home Rd., Marysville, OH 43040, USA; nogay.3@osu.edu

**Keywords:** sow, shoulder lesion, lesion progression, pattern of healing, sow behavior, welfare

## Abstract

**Simple Summary:**

Some sows are known to develop shoulder lesions after giving birth, yet the pattern of development and healing as well as the welfare implications of these lesions is not well-understood. This study found that the size of the lesion when first noticed was related to the duration that the lesion was present and to the maximum size of the lesion before healing. This information has the potential to help guide the treatment of these animals and reduce the severity of their lesions. We monitored these sows throughout lactation and during gestation and found that the only significant behavioral difference between the sows that did and did not have lesions was that the sows without lesions were more likely to change their posture more frequently. We believe this means that more frequent posture changes may have a protective effect against lesion development. Together, the results of this study have added to our understanding of how long it takes these lesions to heal and reveal insight into the lack of behavioral alterations in sows with such lesions.

**Abstract:**

Shoulder lesions can develop in sows during lactation and vary in severity, potentially leading to euthanasia of the sow. There are questions about how these lesions affect the sow’s welfare. Here, sows that were loaded into farrowing pens were monitored prospectively to elucidate the risk for lesion development. To determine whether the presence of shoulder lesions affected behavior, 44 sows with shoulder lesions (LES) were matched to controls (CON) and observed during farrowing for postures and during nursing and gestation for posture and location. Lesions were measured daily. A low weaning body condition score (BCS) increased the possibility of lesion development (OR = 4.8 ± 2.8; *p* = 0.01). There was no difference in behavior between LES and CON with the exception of a higher frequency of postural changes in CON sows (*p* = 0.01). A larger maximum lesion size was associated with larger initial lesion (*p* < 0.01), higher weaning BCS (*p* < 0.001), low parity (*p* < 0.001), and lameness (*p* < 0.001). Median time to healing (24 ± 2.2 days) correlated with maximum lesion size. A low BCS during weaning increased the risk for lesion development and there were multiple factors found which influenced maximum lesion size; however, we did not find behavioral indications that lesions impacted welfare.

## 1. Introduction

Sows developing shoulder lesions, or shoulder sores, after farrowing is a well-known condition that has been reported in up to 50% of animals on some farms [1,2] and is widely considered to be a multifactorial problem. While there appears to be consensus about certain risk factors for lesion development, such as a low body condition score [2,3,4,5], other predisposing factors may include breed, parity, and weaning weight [5]. The development of a shoulder sore can result in economic losses for producers as treatments are limited and sows with severe lesions often have their piglets weaned early, consequently negatively impacting the litter. Sow welfare is also a concern as these lesions are likely painful. 

It is suspected that lesions form in a “top-to-bottom” fashion [6] where superficial tissue damage from prolonged pressure and ischemia progresses into deeper tissue layers, similar to many human pressure sores [7]. Beyond this, however, little is understood about how the lesions progress and heal. Some studies have demonstrated how different treatment choices can alter the rate of healing [5,8], with rubber mat flooring appearing to be an effective feature of treatment plans [4,5]. However, to our knowledge, no studies have examined the daily changes lesions undergo throughout the development and healing process. In order to better implement potential treatment options and improve sow welfare, understanding factors that may affect the severity of lesions in terms of maximum size and lesion duration are essential.

Likewise, while it is suspected that the lesions themselves are painful, particularly when palpated [6], knowledge regarding the behavioral effects of shoulder lesions on sows is limited. Larsen et al. monitored sow behavior for one 24-h observation period two weeks post-farrowing and found sows spent less time lying, more time standing still, and engaged in fewer nursing sessions [9]. Documenting a sow’s behavior throughout the time the wound is present would lend to our understanding of the painfulness of the sores. In particular, discovering whether a sow’s posture, nursing behaviors, and location preferences are affected by the presence of a lesion is essential in evaluating the impact of such lesions on her overall welfare. 

By assessing risk factors for lesion development, compiling data regarding the size of the lesion and the length of time a lesion is present while monitoring the behavior exhibited by a sow over the entirety of the lesion period, we aim to integrate these components and develop a holistic understanding of the impact of the lesions on the sow. It is hypothesized that sows with shoulder lesions will exhibit differences in behavior from similar sows without lesions, and that lesion size and duration will be related to and influenced by factors such as productivity measures and the sow body condition score.

## 2. Materials and Methods

### 2.1. Animals and Housing

The study was conducted at the University of Pennsylvania School of Veterinary Medicine’s Swine Teaching and Research Center, located at the New Bolton Center in Kennett Square, Pennsylvania, between June 2018 and April 2019. The Institutional Care and Use Committee of the University of Pennsylvania reviewed and approved the procedures in this study (IACUC Protocol #804656). A total of 221 Line 241 sows (DNA Genetics, Columbus, NE, USA) in 19 breeding groups (cohorts) of on average 15 sows entered farrowing every other week during the study. Every sow was evaluated at loading into farrowing and weaning and ranged from parity one to nine. Of these 221 sows, 44 developed shoulder lesions (LES) after farrowing. For the behavioral analysis, a control sow (CON) was enrolled for each sow that had a lesion. The remaining sows in the farrowing room with the sow with the shoulder sore were blocked by body condition score, using the visual score described below as well as parity. If a single sow had the same body condition and parity she was enrolled as the control. If multiple sows fit the criteria, a random number generator was used to select one sow. CON sows that later developed a lesion (*n* = 5) were subsequently treated as LES sows and a new CON sow was enrolled using the process above resulting in a total of 22 LES sows and 22 CON sows examined in this study.

Sows entered one of four identical farrowing rooms, each of which contained 10 farrowing pens measuring 2.1 m × 2.0 m and utilized slatted plastic flooring (MIK, International, Ransbach-Baumbach, Germany). Each pen also housed a hinged farrowing crate measuring 0.64 m × 1.73 m which was opened 4 days post-farrowing. At weaning, between 28- and 35-days post-farrowing, sows were moved into individual gestation stalls for breeding by artificial insemination. Sows remained in the stalls for 8 days before being introduced to a single gestation pen with approximately 120 other sows. The gestation pen included nine 3.0 m × 2.1 m solid concrete lying areas, 183.9 m^2^ of slatted concrete flooring, an outdoor concrete patio area measuring 262.6 m^2^, and two deep-bedded straw pits each measuring 4 m × 8.5 m. Sows were fed via two electronic sow feeding stations (Schauer Agrotronic Compident 7, Prambachkirchen, Austria). Sows were observed twice daily post-weaning in the gestation pen until the LES sow’s ulcer had healed completely. If sows developed a shoulder lesion that extended into deeper tissue layers beyond the epidermis they received topical treatment of dilute iodine once or twice daily as needed to prevent secondary bacterial infection. The standard of care also included early weaning for sows with lesions that extended deep into the dermis. These treatment decisions were made on an individual basis, as there is currently no industry-standard grading scale or lesion size cutoff to guide treatment decisions. If a lesion reached the scapular tuber and resulted in exposed bone, the animal would be euthanized. No animals in this study required early weaning or euthanasia due to the severity of a shoulder lesion.

### 2.2. Measurements

#### 2.2.1. Weaning/Loading

Data collected from 221 sows at loading into farrowing included the presence or absence of lameness, body condition score (BCS), and weight. At weaning, the number of days in farrowing, pigs born alive, pigs weaned, and the total litter weight at weaning was recorded where born alive was the number of piglets born in the litter minus the stillborn and mummified piglets, and the parity was the parity post-farrowing. The number of weaned pigs was defined as the number of piglets weaned by that sow. The days in farrowing was the number of days the sow was in the farrowing room from loading to weaning. The wean litter weight was the total weight of all the piglets in the litter at the time of weaning. Body condition score was recorded using a visual scale based on the appearance of the hipbones and spine using the following scale: 1: emaciated; 2: thin; 3: ideal; 4: fat; 5: overly fat [10]. 

#### 2.2.2. Farrowing Behavior

LES and CON sows were video recorded to capture farrowing behavior based on the days post-lesion formation for day 0 (*n* = 37 LES; *n*= 37 CON), day 7 (*n* = 34 LES; *n* = 34 CON), and day 14 (*n* = 16 LES; *n*= 16 CON). Videos were recorded for 1 h using a handheld camera (HandyCam, Sony Inc., Tokyo, Japan) hung 6 m above the farrowing pen to ensure the entire pen was visible. If a sow was weaned prior to the next recording, then no recording was made. Seven sows did not get a day 0 video recorded due to technical issues.

The behaviors that were observed are listed below in Table 1. These included: standing, sitting, and lying posture, which was delineated as the lateral left side, lateral right side, or sternal and which were recorded as durations. Nursing sessions and postural changes were also recorded as counts of each behavior.

#### 2.2.3. Lesion Duration and Size

All sows in farrowing rooms were examined twice daily for the development of shoulder lesions beginning the day after farrowing. A lesion was considered present if the area over the tuber of the scapular spine was reddened and ulceration of the superficial layer of skin was evident. Once a lesion was discovered, it was photographed once daily with a ruler for scale until the lesion was fully healed or the sow was removed from the study for other reasons such as death or illness (*n* = 4). A lesion was determined to be fully healed when the surrounding epidermis was no longer erythematous, there was no active bleeding or serosanguinous drainage, and there was no longer a scab present. Epidermal crusts and scars were deemed an acceptable end point.

Lesions were measured using ImageJ 1.45s freeware (National Institutes of Health, Rockville, MD, USA; http://imagej.net/ImageJ (accessed on 24 June 2021)). The length and width of the lesion were measured three times and the mean was recorded for each. Similarly, the area of the lesion was measured three times and the mean was recorded. 

#### 2.2.4. Gestation Locations

Once sows were introduced to the gestation pen, their location and body posture were recorded twice daily, once in the morning between 06:00 and 8:00 and once in the afternoon between 15:00 and 17:00 by direct observation by a single observer walking through the pen. Location was classified as: on the solid or slatted indoor flooring, in the straw pit, or the outdoor area. Her body position was also recorded, including if she was in an upright position (walking or standing), sitting, or lying (sternal, right lateral, or left lateral). The pairs of sows were monitored until the lesion was fully healed, at which point both animals were no longer part of the study.

### 2.3. Statistical Analysis

#### 2.3.1. Risk of Lesion Development

For data analysis, parity was grouped into 4 categories (1 (*n* = 55), 2 and 3 (*n* = 55), 4 and 5 (*n* = 56), 6 and over (*n* = 55)). 

Stepwise forward and backward estimation was performed for each variable including lameness at weaning and loading, BCS at weaning and loading, weight at weaning and loading, number of days in farrowing, pigs born alive, pigs weaned, parity group, and total litter weight at weaning for impact on lesion development. The value for forward selection was 0.05 and for backward selection was 0.1. Significant factors were included in the final mixed effect model where cohort was included as random effects and BCS at weaning was included as fixed effects.

#### 2.3.2. Farrowing Behavior

Durations were analyzed using mixed-effect generalized linear models with an identity link function and Gaussian distribution. Counts, including nursing and postural changes, were analyzed using mixed-effect Poisson models. Each model included the lesion status (CON or LES) and the day post-lesion development that the video was recorded as fixed effects with the sow as the random effects. *p* values were adjusted for multiple comparisons using a Bonferroni correction. The Wilcoxon rank sum test was used to test for the difference between the proportion of time spent lying on the side with a lesion compared to the time spent lying on the side without the lesion.

#### 2.3.3. Lesion Duration and Size

Lesion duration was analyzed using survival analysis with the number of days until healing used as the end point and sows were censored if they were removed prior to healing (*n* = 4). Factors were included in a univariable cox proportional hazard analysis including parity group, day 1 lesion score, sow loading BCS, sow weaning BCS, sow loading weight, sow weaning weight, number of weaned pigs, number born alive, and wean litter weight. The Spearman rank sum test was used for correlation between lesion duration and maximum lesion size.

Maximum lesion size was modeled using a mixed effect Poisson model. Stepwise forward and backward estimation was performed for each variable including lameness at loading or weaning, BCS at weaning, sow weight at weaning, number of days in farrowing, parity group, and total litter weight at weaning for impact on maximum lesion size. The value for forward selection was 0.05 and for backward selection was 0.10. Significant factors were included in the final mixed effect model where cohort was included as random effects and BCS at weaning, day 1 lesion score, lameness at weaning, litter weight, and parity group as fixed effects. 

#### 2.3.4. Gestation Locations

The proportion of time that sows were found on the solid and slatted floor as well as in the sternal position was normally distributed based on the Shapiro–Wilk test. They were analyzed for differences in mean proportion of observations on solid or slat between LES and CON sows using a 2-sided Student’s *t*-test. The proportion of observations where the sows were found in the straw or up or outside was not normally distributed. They were analyzed with a non-parametric Mann–Whitney U-test to look for differences in medians. Medians and interquartile range (IQR) are presented for data that were not normally distributed. Means and standard deviations (SD) are presented for normally distributed data.

## 3. Results

### 3.1. Risk of Lesion Development

Of the 221 sows enrolled in the study, 20% (*n* = 44) developed a shoulder lesion. BCS of 2 at weaning significantly increased the odds of a sow having a shoulder lesion (OR = 4.8 ± 2.8; *p* = 0.01; CI: 1.5–15.3). None of the other variables had a significant impact on the risk of development of a shoulder lesion (See Table 2).

### 3.2. Farrowing Behavior

There was no significant interaction between the presence of a lesion and day post-lesion, so it was not included in the final model. There was no effect of having a lesion on the duration of time spent sternal, lying laterally, standing, sitting, or number of nursing sessions (see Table 3). There was a significant effect of lesion presence on the number of postural changes with CON sows performing more postural changes (10.2 +/− 1.0) compared to LES sows (7.1 +/− 0.66) (*p* = 0.01). 

There was no difference between the number of minutes a sow with a lesion spent lying on the lesion (7.6 ± 27.4) and lying on the side without the lesion (4.0 ± 19.1) (*p* = 0.47).

### 3.3. Lesion Duration and Size

**Figure 1:**
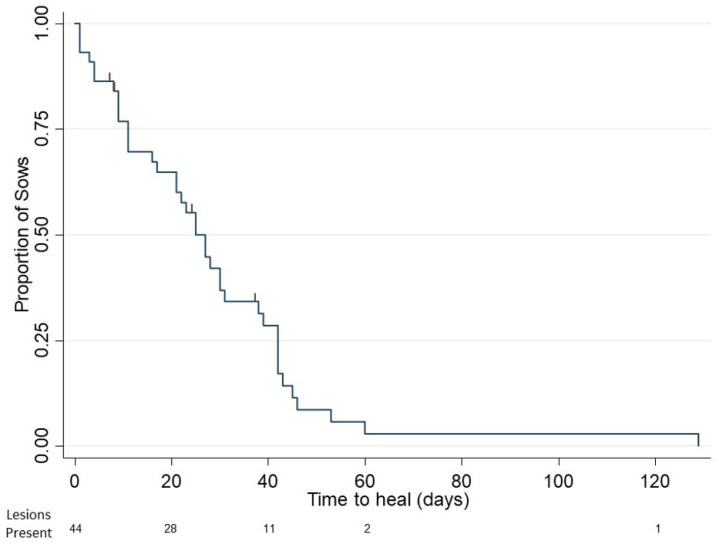
Kaplan–Meier survival estimates illustrating the day at which lesions resolved for 44 sows. Lesions were monitored from day 1 to healing. Healing was defined as the lesion no longer being visible. The median time to healing was 24 days. The number of lesions present at each time point is presented below the *x*-axis. Vertical marks represent sows removed prior to healing (*n* = 4).

There was a moderately significant positive correlation between lesion duration and maximum lesion size (R = 0.70, *p* < 0.0001). The median time until healing was 24 ± 2.2 days, and the range was from 1 day to 129 days (Figure 1). None of the factors examined impacted the median time to healing including sow factors such as BCS, weight, and lameness, or litter factors such as born alive and total pigs weaned (*p* > 0.05).

Predicted maximum lesion size was 10.5 ± 3.2 cm^2^. There was a significant effect of day 1 lesion size on the maximum lesion size (IRR = 1.43 ± 0.13; *p* < 0.001). There was a significant effect of lameness at weaning with lame sows getting larger lesions (28.2 ± 11.8) compared to non-lame sows (10.5 ± 3.7) (*p* < 0.001). Weaning BCS was also significant (*p* < 0.001) with BCS 3 (24.1 ± 9.4) sows and BCS 4 (32.2 ± 11.8) sows having larger lesions than BCS 2 sows (6.6 ± 2.2) (*p* < 0.01). Parity group also had a significant effect on lesion size (*p* < 0.001) where parity group 3 (6.1 ± 2.4) had smaller lesions than parity group 1 (38.5 ± 19.3) (*p* < 0.001) and 2 (40.2 ± 16.3) (*p* < 0.001) as did parity group 4 sows (9.2 ± 3.4) (*p* < 0.01).

### 3.4. Gestation Location and Posture

There were no differences in the proportion of time that sows were found on solid flooring, slatted flooring, in the straw, or outside. There were also no differences in the proportion of observations where the sow was found standing up or in a sternal position (see Table 4).

## 4. Discussion

We found that 20% of the sows enrolled in the study developed a shoulder lesion, which is similar to other studies, ranging from 10% prevalence as reported in Herskin [1], to 48% at peak prevalence in Davies [2], with our study reporting a prevalence intermediate to these. Our finding reinforces that these lesions impact many sows, making understanding their progression and impact on behavior worthwhile. 

Shoulder lesions are a multifactorial disease process, and it can be difficult to predict which sows will develop lesions. This study supports the findings of others where there is a significant correlation between a low body condition score and the development of a shoulder lesion [2,3,4,5]. As BCS decreases, so does the amount of subcutaneous fat that cushions the prominent bony tuber of the scapular spine. Therefore, as a sow lies on her side in lateral recumbency for long periods of time during lactation, the pressure put directly on the scapular spine is focused and increased at that anatomic location, resulting in a greater chance of tissue damage and ischemia at the superficial skin and subcutaneous layers, leading to subsequent ulcer development [6,12]. Managing and maintaining an appropriate BCS in a herd is essential in lessening the incidence of severe shoulder lesions in sows and, therefore, attention should be paid to preventing a low BCS in lactating sows. Other reported risk factors, such as parity and weaned litter weight, have also been reported to increase the odds of a sow developing a lesion [5,12]. However, our study did not find that any significant risk factors associated with sow parity or productivity influenced the risk of lesion development. 

In farrowing, we appreciated a significantly increased frequency of postural changes in CON sows compared to LES sows. Since shoulder lesions are caused by local trauma and ischemia, as described in Dahl-Pedersen [6], sows who are frequently changing posture are less likely to experience prolonged scapular tuber contact with the ground and, therefore, less likely to develop ischemia and subsequent ulcerations. Rolandsdotter [13] supports our findings, stating that sows that exhibited longer uninterrupted bouts of lying behavior on days 0 and 2 post-farrowing were more likely to develop shoulder lesions. Larsen, however, reported an increase in postural changes and restlessness in sows with shoulder lesions and argued that this is a sign of discomfort secondary to the lesions. However, the increase in postural changes in LES sows was not significant in that study and there was no mention of the lengths of uninterrupted bouts of lying time. A study that includes lying behaviors of sows both before and after developing lesions would be necessary to determine whether the frequency of postural changes should be considered as a risk factor for lesion development rather than a sequela.

We did not find any difference in the amount of time LES sows spent lying on the side with a lesion versus the side with no lesion when sows were in farrowing, and this finding is mirrored in other studies [9]. There was also no significant effect of lesion presence on the amount of total time a sow spent lying sternally, lying laterally, standing, sitting, or number of nursing sessions. Interestingly, this finding is not supported by Larsen, where they demonstrated that sows with shoulder lesions spent significantly less time lying in lateral recumbency and less time nursing. In Larsen et al. [9], sows were monitored for a full 24 -h at one common time point, approximately two weeks post-farrowing. It could be that a longer single continuous observation period may have generated such differences. However, we monitored sow behavior until the lesion healed completely, including lactation and in the gestation pen. We found that there was no significant difference in location preference or posture between LES sows and CON sows before or after weaning. Thus, measurements at multiple time points showed no postural differences between LES and CON sows leading to the conclusion that these behavioral measures were not indicative of a welfare impact due to these lesions. Notably, Dahl-Pedersen et al. [6] reported significant pain-related responses to palpation of traumatic neuromas that were likely results of lesions, but there has yet to be any evidence confirming or denying pain associated with the presence of active ulcers. Our findings in this study do not provide sufficient evidence to allow us to deny that sows experience pain associated with active shoulder lesions, but our data does show that behavior associated with posture is not impacted by the presence of a shoulder lesion. 

One aspect of the disease process that has received little attention has been the pattern of progression and healing of these ulcers. In general, the understanding is that lesions are likely to develop within a few days to weeks after farrowing and will quickly heal after weaning. However, few studies have followed lesions throughout their entire progression, and even fewer have followed lesions until they have healed completely [2,5]. By measuring lesions from the day they developed until the day they healed, we were able to obtain a more comprehensive picture of the pattern of progression and healing that a shoulder lesion follows. We found that the median number of days a lesion was present, from initial appearance until healing, was 24 ± 2.2 days, with a range from one to 129 days. Zurbrigg et al. [5] reported an average healing time of 32 days if sows received no treatment (rubber mats or stainless-steel plates), 25 days with rubber mat treatment, and 39 days with stainless steel treatment. Meanwhile, Davies et al. [2] reported a maximum healing time of 68 days. While the median time to healing in our study is on the lower end of what has been reported, 129 days to heal is quite long, and similar lengths have not been reported in other studies. We did not find that any of the factors studied here had an impact on the time it took for a lesion to heal, though a longer duration was correlated with larger maximum lesion size. 

We determined that the size of the lesion on the day it was first discovered was significantly correlated to the maximum size of the lesion. Kaiser et al. [8] reported similar findings, stating that the size of a lesion on day 1 was significantly associated with the size of the lesion on day 14 and day 21. Our data also showed that lower parity animals had larger lesions, as did sows with a BCS 3 or 4 compared to sows with a BCS of 2. Sows that were lame also had larger lesions. These findings suggest that a larger sow with a lesion that puts more weight on her shoulder is likely to develop a lesion that is significantly larger in area. Likewise, lameness may predispose a sow to decrease her time spent moving, leading to prolonged pressure on the shoulder and an increase in lesion size. In opposition to our findings, Davies tended to see that a decrease in BCS and an increase in parity resulted in more severe lesions [2]. However, this data was not always significant or consistent, and he discussed the importance of freedom of movement, scapular tuber depth, and other potential environmental factors contributing to the severity of shoulder lesions [2]. Our findings further confirm that BCS is critical in decreasing the risk of lesions but also indicate that lesion size can be influenced by maintaining a healthy BCS in lactating sows and not allowing them to become over-conditioned. Future studies should prospectively examine the relationship between postural changes and lesion development, which could lead to methods of prevention, as well as other possible measures of welfare such as cognitive bias, which could give more insight into the affective state of sows with shoulder lesions.

## 5. Conclusions

We found that the presence of shoulder lesions did not significantly alter a sow’s behavior, as other studies have also shown [5,6,9]. We concluded that the frequency of postural changes was significantly greater in sows without a lesion and, thus, more frequent postural changes may protect against shoulder lesion development. While we cannot state that the LES sows did not feel pain associated with the lesions based on the lack of behavioral differences from the controls, we did not find evidence that sows with lesions changed their routine behaviors such as lying side, posture, or number of nursing sessions. The duration of a lesion was correlated with maximum lesion size, and maximum lesion size was influenced by the size of the lesion on day 1, a high BCS, low parity, and lameness, all leading us to conclude that larger animals that remain lying in one position for long periods of time are more likely to develop more severe lesions.

## Figures and Tables

**Table 1:** Ethogram, outlining sow behaviors in farrowing and their respective definitions.

Behavior	Definition
sternal recumbency	Sow lying with entire ventrum in contact with the floor in a resting position; there is no scapular contact with the floor.
standing	Sow has the soles of all four feet on the ground and legs are fully extended; includes ambulating.
sitting	Hind end and rump are in contact with the ground with hind legs tucked underneath; front legs straightened and soles of front feet are in contact with the ground, supporting the cranial half of the body.
lateral recumbency	Sow is on her side with the, scapula and caudal half of the body touching the floor and all legs visible. ^1^
posture changes	Any change from one posture to another.
session of nursing	At least half of the litter begins nursing until at least half of the litter stops nursing.

^1^ Cui et al., 2011 [11].

**Table 2:** Descriptive statistics for sow loading data, litter data, and sow weaning data for sows that developed a shoulder lesion (LES) and control sows (CON). Results are presented as mean plus or minus standard deviation. Sows were weighed when loaded into farrowing at day 109 of gestation and given a body condition score at that time. Born alive refers to the number of piglets born in the litter minus those that were stillborn or mummified. The parity is the parity post-farrowing. Number of weaned pigs is defined as the number of piglets weaned by that sow. Days in farrowing is the total time that the sow was in the farrowing room from loading to weaning. The wean litter weight refers to the total weight of all the piglets in the litter at weaning.

	CON (*n* = 176)	LES (*n* = 44)
Sow load weight (kg)	255.0 ± 43.1	240.9 ± 39.6
BCS at loading	3.2 ± 0.5	3.1 ± 0.5
Lameness at loading (%)	16.4%	22.7%
Parity (#)	3.8 ± 2.2	3.1 ± 2.0
Born alive (#)	15.3 ± 4.0	15.7 ± 4.1
Days in farrowing (#)	39.9 ± 1.8	40.0 ± 1.6
Wean litter weight (kg)	44.3 ± 10.8	47.8 ± 11.5
Weaned pigs (#)	11.1 ± 2.5	11.8 ± 2.7
Lameness at weaning (%)	15.3%	20.4%
Sow wean weight (kg)	235.9 ± 48.4	218.8 ± 45.4

**Table 3:** Median duration (minutes) of each behavior plus or minus IQR for cases, those sows that developed a lesion (LES) and their matched control (CON) as well as the median number of nursing sessions. Sows were recorded on days 0, 7, and 14 post-lesion development for as long as they remained in farrowing. Each video was 1 h in duration.

	CON	LES
Sternal	18.3 ± 18.1	15.9 ± 22.3
Stand	6.7 ± 11.8	8.1 ± 17.2
Sit	0.6 ± 3.0	0.7 ± 2.6
Lateral	30.3 ± 28.4	24.3 ± 34.6
Nursing sessions	1.0 ± 1.0	1.0 ± 1.0

**Table 4:** Proportion of observations where sows with a shoulder sore (LES) and control sows (CON) were observed on different flooring types, in different areas in the gestation pen, in a sternal position, or standing up at the time of the observation. Sows were observed twice daily after 8 days post-weaning once they were placed in the gestation pen post-breeding. Proportions are means and standard deviations for slatted and solid flooring and are otherwise medians and interquartile ranges.

Location or Posture	CON (*n* = 24)	LES (*n* = 24)	*p*-Value
Slatted	0.55 ± 0.06	0.58 ± 0.05	0.71
Solid	0.43 ± 0.06	0.42 ± 0.05	0.91
Outside	0.04 ± 0.36	0.06 ± 0.44	0.80
Straw	0.00 ± 0.19	0.08 ± 0.25	0.11
Sternal	0.38 ± 0.31	0.37 ± 0.25	0.88
Up	0.29 ± 0.29	0.33 ± 0.34	0.48

## Data Availability

Not applicable.
